# Melanoma of Unknown Primary: Evaluation of the Characteristics, Treatment Strategies, Prognostic Factors in a Monocentric Retrospective Study

**DOI:** 10.3389/fonc.2021.627527

**Published:** 2021-03-05

**Authors:** Paolo Del Fiore, Marco Rastrelli, Luigi Dall’Olmo, Francesco Cavallin, Rocco Cappellesso, Antonella Vecchiato, Alessandra Buja, Romina Spina, Alessandro Parisi, Renzo Mazzarotto, Beatrice Ferrazzi, Andrea Grego, Alessio Rotondi, Clara Benna, Saveria Tropea, Francesco Russano, Angela Filoni, Franco Bassetto, Angelo Paolo Dei Tos, Mauro Alaibac, Carlo Riccardo Rossi, Jacopo Pigozzo, Vanna Chiarion Sileni, Simone Mocellin

**Affiliations:** ^1^ Surgical Oncology Unit, Veneto Institute of Oncology IOV - IRCCS, Padua, Italy; ^2^ Department of Surgery, Oncology and Gastroenterology (DISCOG), University of Padua, Padua, Italy; ^3^ Emergency Department- Azienda Ospedaliera Padova, Padova, Italy; ^4^ Independent Statistician, Solagna, Italy; ^5^ Surgical Pathology and Cytopathology Unit, Department of Medicine (DIMED), University of Padua, Padua, Italy; ^6^ Department of Cardiological, Thoracic, Vascular Sciences and Public Health, University of Padua, Padua, Italy; ^7^ Radiotherapy Unit, Veneto Institute of Oncology, IOV-IRCCS, Padua, Italy; ^8^ Department of Radiotherapy, Ospedale Civile Maggiore, Azienda Ospedaliera Universitaria Integrata, Verona, Italy; ^9^ Postgraduate School of Occupational Medicine, University of Verona, Verona, Italy; ^10^ Department of Medicine (DIMED), University of Padua, Padua, Italy; ^11^ Clinic of Plastic Surgery, Department of Neuroscience, Padua University Hospital, University of Padua, Padua, Italy; ^12^ Unit of Dermatology, University of Padua, Padua, Italy; ^13^ Melanoma Oncology Unit, Veneto Institute of Oncology IOV-IRCCS, Padua, Italy

**Keywords:** melanoma of unknown primary, occult primary melanoma, skin cancer, melanoma, MUP, melanoma treatment, immunotherapy, target therapy

## Abstract

**Background:**

Melanoma of unknown primary (MUP), accounts for up to 3% of all melanomas and consists of a histologically confirmed melanoma metastasis to either lymph nodes, (sub)cutaneous tissue, or visceral sites without any evidence of a primary cutaneous, ocular, or mucosal melanoma. This study aimed to investigate the characteristics, treatment strategies, and prognostic factors of MUP patients, in order to shed some light on the clinical behavior of this malignancy.

**Methods:**

All the consecutive patients with a diagnosis of MUP referring to our institutions between 1985 and 2018 were considered in this retrospective cohort study. The records of 173 patients with a suspected diagnosis of MUP were retrospectively evaluated for inclusion in the study. Patient selection was performed according to the Das Gupta criteria, and a total of 127 MUP patients were finally included in the study, representing 2.7% of the patients diagnosed with melanoma skin cancer at our institutions during the same study period. A second cohort of all consecutive 417 MKP patients with AJCC stages IIIB–IV, referring tions in the period considered (1985–2018), was included in the study to compare survival between MUP and MKP patients. All the diagnoses were based on histopathologic, cytologic and immunohistochemical examination of the metastases. All tumors were re-staged according to the 2018 American Joint Committee on Cancer (AJCC) 8^th^ Edition.

**Results:**

Median follow-up was 32 months (IQR: 15–84). 3-year progression-free survival (PFS) was 54%, while 3-year overall survival (OS) was 62%. Worse OS and PFS were associated with older age (P = 0.0001 for OS; P = 0.008 for PFS), stage IV (P < 0.0001 for OS; P = 0.0001 for PFS) and higher Charlson Comorbidity Index (P < 0.0001 for OS and P = 0.01 for PFS). Patients with lymph node disease showed longer PFS (P = 0.001) and OS (P = 0.0008) than those with (sub)cutis disease. Complete lymph node dissection (CLND) was the most common surgical treatment; a worse OS in these patients was associated with the number of positive lymph nodes (P = 0.01), without significant association with the number of retrieved lymph nodes (P = 0.79). Survival rates were lower in patients undergoing chemotherapy (CT) and target therapy (TT), and higher in those receiving immunotherapy (IT). 417 patients with AJCC stages IIIB–IV of Melanoma Known Primary (MKP) were included for the survival comparison with MUP. 3-year PFS rates were 54 and 58% in MUP and MKP, respectively (P = 0.30); 3-year OS rates were 62 and 70% in MUP and MKP, respectively (P = 0.40).

**Conclusions:**

The most common clinical scenario of our series was a male patient around 59 years with lymph node disease. We report that CLND associated with IT was the best treatment in terms of survival outcome. In the current era of IT and TT for melanoma, new studies have to clarify the impact of novel drugs on MUP.

## Introduction

Melanoma of unknown primary (MUP) also known as occult primary melanoma accounts for up to 3% of all melanomas (1) and consists of a histologically confirmed melanoma metastasis to either lymph nodes, (sub)cutaneous tissue, or visceral sites. The diagnosis of MUP is definitive when a primary cutaneous, ocular, or mucosal melanoma is missing after a thorough physical examination and histological revision of previously excised melanocytic lesions. In 1963, Das Gupta and collaborators defined the diagnostic criteria for MUP (2). Such criteria exclude patients who do not receive complete physical examination (including anus/genitalia and ophthalmological visit); those with evidence of previous orbital enucleation, those without histological documentation of prior surgical or non-surgical procedures (*e.g.*, for a mole, birthmark, freckle, chronic paronychia, or skin blemish), and those with nodal involvement and presence of a scar in the skin area drained by the lymphatic basin (2). Of note, according to Kamposioras, only 16% of publications on MUP applied the stringent Das Gupta’s exclusion criteria, thus the remaining might have included as MUP some melanoma of known primary (MKP) (3). The peak incidence of MUP occurs between the fourth and fifth decade of age, which is comparable to that of MKP of the skin but earlier than those arising from the mucosa. MUP is also more common in men than women. The management of patients with MUP has been the same to the management of patients with metastatic melanoma and with MKP. Although the survival of patients with stage III−IV MUP as compared to patients with stage III−IV MKP has been richly explained (4–6) including the hypotheses attributable to immune-mediated control of the primary tumor in patients with MUP, a distinct signature of MUP that differentiate the treatment strategies for MUP and MKP has not been defined. To do this, more retrospective cohort studies such as ours are needed to compare outcomes between patients with MUP and stage-matched MKP during novel therapy.

This study aimed to investigate the characteristics, treatment strategies and prognostic factors of MUP patients, in order to shed some light on the clinical behavior of this rare type of melanoma. In addition, survival in MUP patients was compared with survival in MKP patients with the same stage and metastatic sites. The clinical impact of our study is to build a retrospective cohort study for the clinical features and behavior of MUP in the evolving era of immunotherapy, targeted therapies, and their combinations.

## Materials and Methods

### Study Design

All the consecutive patients with a diagnosis of MUP referring to the Melanoma and Sarcoma Clinic of the Veneto Institute of Oncology (IOV) and the Department of Surgery Oncology and Gastroenterology (DISCOG) of the University of Padua (Italy) between 1985 and 2018 were considered in this retrospective cohort study. IOV and DISCOG are level III referral institutions in Northeastern Italy. Most patients are referred for diagnosis and/or first-line treatment, while some patients are referred for disease progression after being treated in level I–II centers. The study was conducted according to the Helsinki Declaration principles and was approved by the local Ethics Committee (17/04/2020, approval No. 7254). All patients gave their consent for data collection and analysis for scientific purposes.

### Patients

The records of 173 patients with a suspected diagnosis of MUP referring to IOV or DISCOG between 1985 and 2018 were retrospectively evaluated for inclusion in the study.

Patient selection was performed according to the Das Gupta criteria (2) (**Table 1**). Forty-six patients were excluded because of unclear information on primary melanoma (14 patients), misdiagnosis of MUP (medical history of previous cutaneous melanoma, 11 patients) or “evidence of previous skin excision or other surgical manipulation of a mole, freckle, birthmark, paronychia or skin blemish”, or “evidence of metastatic melanoma in a draining lymph node with a scar in the area of skin supplying the lymph node basin” (21 patients) (1). A total of 127 MUP patients were finally included in the study, representing 2.7% of the patients diagnosed with melanoma skin cancer (127 out of 4,703 patients) at our institutions during the same study period.

**Table 1:** Das Gupta’s exclusion criteria.

Das Gupta’s exclusion criteria
Evidence of previous orbital exenteration or enucleation
Evidence of previous skin excision,electrodessication, cauterization or other surgical manipulation of a mole, freckle,birthmark, paronychia, or skin blemish.
Evidence of metastatic melanoma in a draining lymph node with a scar in the area of skin supplying that lymph node basin.
Lack of a nonthorough physical examination, including the absence of an ophthalmologic, anal, and genital exam.

A second cohort of all consecutive 417 MKP patients with AJCC stages IIIB–IV, referring to our institutions in the period considered (1985–2018), was included in the study to compare survival between MUP and MKP patients.

### Diagnosis and Treatment

All the diagnoses were based on histopathologic, cytologic, and immunohistochemical examination of the metastases. All tumors were re-staged according to the 2018 American Joint Committee on Cancer (AJCC) 8^th^ Edition—TNM staging system (7) was used for tumor staging.

Patients with melanoma metastases in the (sub)cutis, soft tissue, and/or lymph nodes, without a detectable primary tumor were diagnosed s stage III disease, while those with distant metastases including visceral metastases are diagnosed as stage IV.

The surgical treatment included wide resection (WR) in patients with (sub)cutis/soft tissue lesion, complete lymph node dissection (CLND) in those with lymph node metastasis and metastasectomy in those with complete, resectable distant/visceral location.

Radiation therapy (RT) was performed according to location, stage, surgical radicality, and residual disease load. Medical oncology treatments included target therapy (TT), immunotherapy (IT), and classic chemotherapy (CT). In some patients, electrochemotherapy (ECT) and hyperthermic limb perfusion (ILP) were also employed.

IT with high-dose interferon (IFN HD) was used as adjuvant treatment after radical surgery in stage III patients. Since 2012, stage IV patients were treated with targeted therapy (TT) if the melanoma carried the V600E BRAF mutation: in particular, the combination of BRAF and MEK inhibitors (Dabrafenib and Trametinib or Vemurafenib and Cobimetinib, respectively); in case of *BRAF* wild type disease, immune checkpoint blockade with anti-PD1 monoclonal antibodies (Pembrolizumab or Nivolumab) alone or in combination with anti-CTLA4 monoclonal antibodies (Ipilimumab) (8, 9).

Systemic CT (*i.e.* dacarbazine and bio-chemotherapy regimens) was administered before 2012.

Follow-up was performed every three months for the first two years, then every six months up to the 5^th^ year, and once a year thereafter. Disease progression was defined as local disease recurrence, lymph node metastasis and/or distant metastasis.

### Data Collection

All data were extracted from a prospectively maintained database. Demographics included age at diagnosis, gender and family history of cancer, while melanoma-related information included clinical presentation, metastasis size, and AJCC TNM stage (7). Tumor stage according to Balch’s proposal (which includes stage IV non-visceral tumors in stage III) was also assessed (10). Comorbidity status was summarized using the age-adjusted Charlson Comorbidity Index (11). Neoplastic comorbidity and autoimmune comorbidity were evaluated separately. Information on treatment strategy included surgical therapy (WR, CLND, metastasectomy) and medical therapy (radiotherapy, target therapy, immunotherapy and chemotherapy). Follow-up information was extracted from the reports of scheduled visits. Overall survival was calculated from diagnosis to death (by any cause) or to the last visit, while recurrence/progression-free survival was calculated from diagnosis to recurrence/progression or to the last visit.

### Statistical Analysis

Categorical data were summarized as frequency and percentage, while continuous data as median and interquartile range (IQR).

Survival curves were calculated using Kaplan–Meier method. Survival estimates were compared between MUP and MKP patients using the log-rank test.

The association between clinically relevant variables and survival was assessed using Cox regression models. Effects sizes were reported as hazard ratio (HR) with 95 per cent confidence interval (95% CI). Of note, the association between surgical treatments and survival was not evaluated because surgical treatments mirrored the clinical presentation of MUP.

Multivariable analysis of survival was performed with Cox regression models including a set of clinically relevant factors at diagnosis (*i.e*. age, Charlson Comorbidity Index, and tumor presentation). Metastasis size was not included in the analysis because this information was available only for lymph node metastases (but not skin metastases). In addition, some potential factors could not be included in the multivariable models due to collinearity with presentation (AJCC stage), rarity of the events (neoplastic and autoimmune comorbidity) or incomplete information (BRAF mutational status).

The association between medical treatments and tumor stage was evaluated using Fisher’s exact test.

All tests were two-sided and a p-value less than 0.05 was considered statistically significant. Statistical analysis was performed using R 4.0 (R Foundation for Statistical Computing, Vienna, Austria) (12).

## Results

### Patients

Of the 173 patients with MUP considered in this study, 46 were excluded, according to the Gupta’s criteria. One hundred and twenty-seven patients (78 males and 49 females; median age 59 years) with a diagnosis of MUP between 1985 and 2018 were included in the analysis. Patient and tumor characteristics are shown in **Table 2**. There were 68 AJCC stage III tumors (Balch stage III) and 59 AJCC stage IV tumors, of whom 25 were non-visceral tumors (Balch stage III) and 34 were visceral tumors (Balch stage IV). *BRAF* was mutated in 38 out of 68 evaluable patients (56%).

**Table 2:** Patient and tumor characteristics.

Variable			AJCC stage III	AJCC stage IV	
			Patient with lymph node metastases	Patient with (Sub)cutis metastases	Patient with visceral metastases
	N patients:	127	68	25	34
Demographics	Age at diagnosis, year*^a^*	59 (48–70)	57 (47–67)	60 (48–69)	62 (49–73)
	Sex:				
	Female	49 (39)	24 (35)	12 (48)	13 (38)
	Male	78 (61)	44 (65)	13 (52)	21 (62)
	Family history of cancer*^b^*	11 (12)	4 (8)	3 (19)	4 (15)
Tumor characteristics	Size of lymph node metastasis, cm*^a^*,*^c^*	4.0 (2.5–5.0)	4.0 (2.5–5.0)	–	4.0 (3.4–6.0)
	AJCC stage:				
	III	68 (54)	68 (100)	0	0
	IV	59 (46)	0	25 (100)	34 (100)
Comorbidity status	Charlson Comorbidity Index*^a^*	2 (1–3)	2 (0–3)	2 (1–3)	2 (1–4)
	Neoplastic comorbidity	19 (15)	10 (15)	2 (8)	7 (21)
	Autoimmune comorbidity	22 (17)	9 (13)	3 (12)	10 (29)

Data expressed as n (%) or ^a^median (IQR). Data not available in ^b^one and ^c^29 patients.

### Treatment

**Figure 1:**
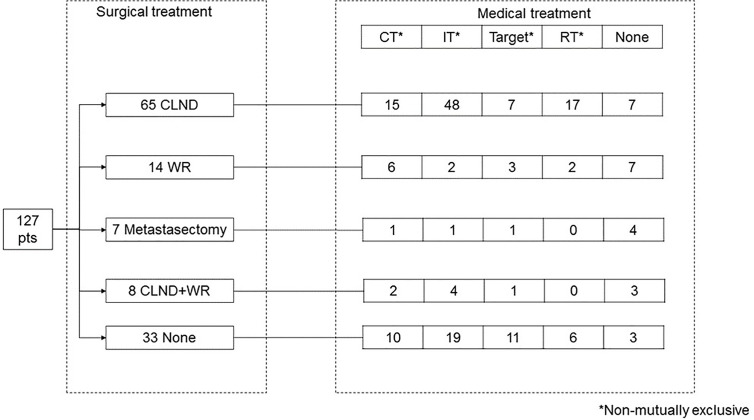
Surgical and medical treatment.

Treatment strategies are shown in **Figure 1**. Ninety-four patients (74%) underwent surgical treatment: 65 CLND, 14 WR, seven metastasectomy, and eight CLND+WR, while 30 patients underwent only medical treatment and three refused the treatment. CLND was performed in axilla (27 patients), groin (eight patients) or neck (12 patients), with a median of 23 retrieved nodes (IQR 18–32) and a median of two positive nodes (IQR 1–5). Such information was not available for six patients.

Medical treatment was administered to 103 patients (81%), with 38 patients receiving more than one treatment, and 65 patients receiving only one treatment. Overall, 34 patients received chemotherapy, which was more frequent among stage IV patients (37 *vs*. 18% in stage III patients, p = 0.02). Seventy-four patients received immunotherapy, which was more frequent among stage III patients (72 *vs*. 42% in IV patients, p = 0.001). Target therapy was administered to 23 patients, with no statistically significant difference between stage III *vs*. IV patients (13 *vs*. 23%, p = 0.19). Twenty-five patients (20%) received radiotherapy, with no statistically significant difference between stage III *vs*. IV patients (23 *vs*. 15%, p = 0.34). Nine patients received chemo-radiotherapy.

### Survival

Median follow-up was 32 months (IQR 15–84). At the analysis, seven patients had local recurrence, 39 had recurrence with clinical upstaging, and 19 had disease progression.

**Figure 2:**
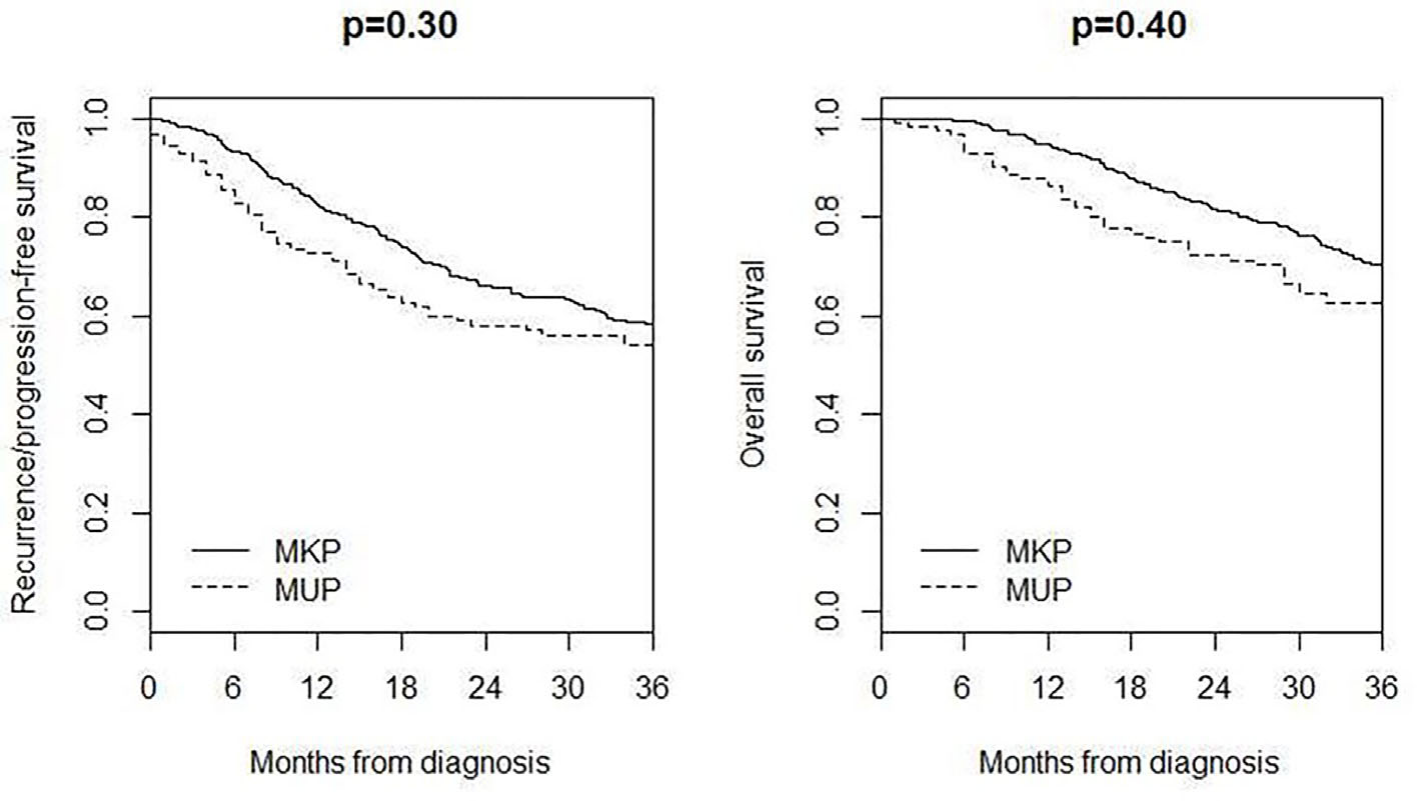
Overall survival and recurrence/progression-free survival in MUP and MKP patients.

3-year recurrence/progression-free survival was 54%, while 3-year overall survival was 62% (**Figure 2**).

Univariate analyses of recurrence/progression-free survival and overall survival are reported in **Table 3**. Impaired recurrence/progression-free survival was associated with older age (HR 1.03, 95% CI 1.01 to 1.04; p = 0.008), stage IV (HR 2.77, 95% CI 1.66 to 4.63; p = 0.0001) and higher Charlson Comorbidity Index (HR 1.16, 95% CI 1.03 to 1.30; p = 0.01). Patients with lymph node metastasis showed longer recurrence/progression-free survival than those with (sub)cutis metastases (HR 0.37, 9%% CI 0.20 to 0.68; p = 0.002). Among patients who underwent RLND, overall survival was associated with the number of positive lymph nodes (HR 1.06, 95% CI 1.01 to 1.11; p = 0.01) but not with the number of retrieved nodes (HR 1.00, 95% CI 0.96 to 1.03; p = 0.79). Impaired overall survival was associated with older age (HR 1.04, 95% CI 1.02 to 1.06; p = 0.0001), stage IV (HR 3.43, 95% CI 2.00 to 5.89; p < 0.0001) and higher Charlson Comorbidity Index (HR 1.25, 95% CI 1.12 to 1.40; p < 0.0001). Patients with lymph node metastasis showed longer overall survival than those with (sub)cutis metastases (HR 0.34, 9%% CI 0.18 to 0.65; p = 0.001). Among patients who underwent CLND, overall survival was associated with the number of positive lymph nodes (HR 1.06, 95% CI 1.01 to 1.11; p = 0.01) but not with the number of retrieved nodes (HR 1.00, 95% CI 0.96 to 1.03; p = 0.79).

**Table 3:** Univariate analysis of survival.

Variable	Recurrence/progression-free survival		Overall survival	
	HR (95% CI)	p-value	HR (95% CI)	p-value
Age at diagnosis, years:	1.03 (1.01 to 1.04)	0.008	1.04 (1.02 to 1.06)	0.0001
Sex:				
Female	Reference	–	Reference	–
Male	1.05 (0.63 to 1.75)	0.86	1.37 (0.79 to 2.36)	0.26
Family history of cancer:				
No	Reference	–	Reference	–
Yes	2.10 (0.97 to 4.51)	0.06	1.52 (0.64 to 3.62)	0.34
Size of lymph node metastasis, cm*^a^*	1.09 (0.97 to 1.22)	0.15	1.09 (0.94 to 1.26)	0.27
AJCC stage:				
III	Reference	–	Reference	–
IV	2.77 (1.66 to 4.63)	0.0001	3.43 (2.00 to 5.89)	<0.0001
Charlson Comorbidity Index	1.16 (1.03 to 1.30)	0.01	1.25 (1.12 to 1.40)	<0.0001
Presentation:				
(Sub)cutis metastases	Reference	–	Reference	–
Lymph node metastases	0.37 (0.20 to 0.68)	0.002	0.34 (0.18 to 0.65)	0.001
Visceral metastases	1.03 (0.54 to 1.96)	0.94	1.36 (0.71 to 2.62)	0.36
Neoplastic comorbidity:				
No	Reference	–	Reference	–
Yes	1.40 (0.71 to 2.74)	0.34	1.73 (0.90 to 3.35)	0.10
Autoimmune comorbidity				
No	Reference	–	Reference	–
Yes	1.23 (0.64 to 2.37)	0.53	1.13 (0.57 to 2.23)	0.73
*BRAF*:				
Wild Type	Reference	–	Reference	–
Mutation	1.22 (0.65 to 2.29)	0.54	0.71 (0.35 to 1.43)	0.34
CT:				
No	Reference	–	Reference	–
Yes	2.76 (1.66 to 4.57)	<0.0001	2.23 (1.33 to 3.75)	0.002
Immune therapy:				
No	Reference	–	Reference	–
Yes	0.58 (0.35 to 0.95)	0.03	0.53 (0.32 to 0.89)	0.02
Target therapy:				
No	Reference	–	Reference	–
Yes	3.37 (1.94 to 5.87)	<0.0001	1.85 (1.01 to 3.40)	0.04
RT:				
No	Reference	–	Reference	–
Yes	1.35 (0.6 to 2.42)	0.31	1.13 (0.61 to 2.09)	0.71

a Among patients with lymph node metastases or visceral metastases.

Of note, survival was impaired in patients undergoing CT and target therapy and improved in those receiving immune therapy (**Table 3**).

Multivariable analysis identified only stage as independent predictor of survival among clinically relevant factors at diagnosis (**Table 4**). Patients with lymph node metastases had longer recurrence/progression-free survival (HR 0.36, 95% CI 0.19 to 0.67; p = 0.001) and overall survival (HR 0.33, 9%% CI 0.17 to 0.63; p = 0.0008) than those with (sub)cutis metastases.

**Table 4:** Multivariable analysis of overall survival.

Variable	Recurrence/progression-free survival		Overall survival	
	HR (95% CI)	p-value	HR (95% CI)	p-value
Age at diagnosis, years:	1.01 (0.98 to 1.04)	0.42	1.03 (0.99 to 1.06)	0.11
Charlson Comorbidity Index	1.09 (0.89 to 1.32)	0.41	1.10 (0.91 to 1.33)	0.31
Presentation:				
(Sub)cutis metastases:	Reference	–	Reference	–
Lymph node metastases:	0.36 (0.19 to 0.67)	0.001	0.33 (0.17 to 0.63)	0.0008
Visceral metastases:	0.94 (0.49 to 1.80)	0.85	1.12 (0.89 to 2.17)	0.73

### Comparison of Survival in MUP and MKP Patients

**Figure 3:**
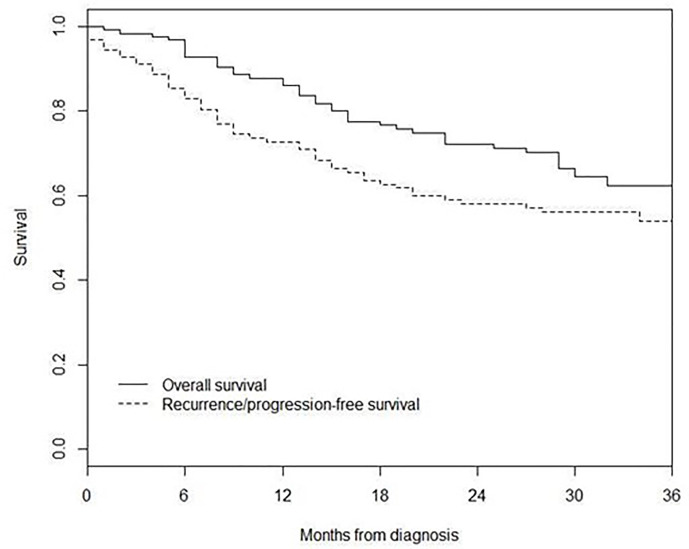
Overall survival and recurrence/progression-free survival.

Four hundred and seventeen MKP patients (213 males and 204 females; median age 59 years, IQR 45–70) with AJCC stage IIIB–IV were included in the comparison of survival, 3-year recurrence/progression-free survival was 54% in MUP and 58% in MKP (p = 0.30), and 3-year overall survival was 62% in MUP and 70% in MKP (p = 0.40) (**Figure 3**).

## Discussion

This study describes patient characteristics, therapeutic approaches, and prognosis of a series of 127 consecutive cases of melanoma of unknown primary (MUP).

The most common clinical scenario in this cohort was a male patient with a median age of 59 years, presenting with a melanoma localized at lymph nodes with neither a detectable primary tumor nor a history of previous melanoma removal, and satisfying all the Das Gupta’s exclusion criteria for the definition of MUP.

The median size of lymph node involvement was 4 cm, irrespective of AJCC III or IV stage (*i.e.* with no difference in size between patients with nodal metastases alone, and those with concurrent nodal and visceral metastases). CLND was the most common surgical treatment, and the survival was associated with the number of positive lymph nodes, without significant association with the number of retrieved lymph nodes, in agreement with other studies (13–16). As expected, our results show a worse survival for advanced stage of disease. Considering the staging, our data support AJCC staging system and suggest that the Balch proposal to consider subcutaneous disease as stage III could be not appropriate. In fact, in our series, patients with subcutaneous disease (AJCC stage IV, Balch stage III) had a worse survival than those with lymph nodes metastases (AJCC stage III, Balch stage III), supporting the inclusion of patients with subcutaneous metastases alone in AJCC stage IV.

In addition, the Charlson comorbidity status resulted to be associated with a worse survival in our series.

Considering stage and treatment of MUP, two milestones have been reported. In 2006, the routine use of combined PET/CT at diagnosis in MUP patients increased the shift from stage III to stage IV, and starting from 2011 the introduction of immune and targeted therapy changed the clinical outcome and long-term survival in advanced melanoma. However, in our center, as well in Italy, both therapies were available only in CRTs till 2014; therefore their impact in this series is limited at the last five years. In this historical context, a possible limitation of our study is the long period considered and the imaging and therapeutic changes introduced. Nevertheless, even pooling and considering as “immune-therapy” (IT) the classical interferon option and the novel immune-modulating opportunities (*i.e*. CTLA4 inhibitors and PDL1 inhibitors), IT was the medical treatment associated with the best survival outcome. The lower survival obtained in patients treated with traditional chemotherapy (CT) was in line with the significant superiority of IT compared to CT in all clinical studies. The lower effect of targeted therapy (TT) was due to selection or to more aggressive features in BRAF mutated patients, or could be related to the immune mechanism involved in the initial elimination of melanoma. Indeed a MUP could be considered a recurrence of an immune eliminated melanoma, and IT could restore an effective immune response, and a greater effect of IT in patients with a “fable immunity” was often observed and reported in the literature in old patients and in immune deficient patients. The comparison of IT to TT in this type of melanoma should be tested in large cohorts and prospectively.

Additionally, the origin of MUP is still an open question, and future studies elucidate whether MUP has to be considered and treated as a melanoma with a known primary (MKP) or represents a different entity. As for survival, we could not demonstrate a difference among MUP and MKP as already reported by other groups. However many authors showed a significant improved survival of MUP compared with MKP (3–5, 17–22).

This was originally explained by Smith and Stehlin in 1965 with a phenomenon of immunological *spontaneous regression* of the primitive tumor (T of TNM). Of note, in contrast to this interpretation, a partial regression of the primary tumor at dermatoscopy has traditionally been recognized as a negative prognostic sign. Therefore linking *regression* to better survival seems at least in part a contradiction, as for melanoma. However the explanation by Smith and Stehlin has been re-proposed by many authors afterwards and is cited also by Anbari and coworkers in 1997 alongside with other criteria of exclusion of MUP (*i.e. a concurrent, unrecognized melanoma or a previously excised, misdiagnosed melanoma*). Indeed, the original contribution of the latter report at the end of last century was the proposal of a new explanation for the origin of MUP: it could represent a primary tumor (T of TNM) within a node rather than a metastatic process to the regional basin (N of TNM). This could explain the better prognosis of MUP patients when compared to MKP, but this does not explain subcutaneous metastases without nodes or visceral metastasis only.

Whatever the origin, it should be considered that the absence of cutaneous/mucosal malignancy in MUP patients could explain by itself their better prognosis for the lesser tumor load (*i.e*. lower amount of cancer stem cells able to metastasize and/or give rise to recurrent disease).

Recently, new reports tried to assess the existence of any correlation between mutations in the main genes (BRAF/NRAS) involved in melanoma initiation and progression (23); they have proposed a distinct molecular classification for MUP to explain the differences in patient outcomes. MUP patients presents consistently BRAF and TERT promoter mutations, suggesting a cutaneous origin. BRAF mutations rate in MUPs appears similar to MKPs; however, for MUPs the rate for V600K seems higher than the rate for MKPs (24). Melanomas with the V600K mutation are characterized by a lower dependence on the activation of the ERK pathway and greater use of alternative pathways; against these melanomas they have a higher mutational load and respond better to immunotherapy; this would concretely explain the better response to immunotherapy and the worse response to BRAFi/MEKi of the MUPs (25–27). The strengths of our study include the diagnosis of MUP based on Das Gupta’s criteria (2), the sample size (one of the largest in MUP literature), the evaluation of Balch’s staging proposal, and the evaluation of systemic treatments.

The present study has also some limitations. First, it is a single-center study, thus the generalizability of the findings is limited. Second, the retrospective nature of the study limited the availability of data (*e.g.* mutational status). Third, the included patients were treated with heterogeneous modalities because of the long period of inclusion. Fourth, the new medical options now available both in in the adjuvant as in the metastatic setting for all patients could make the distinction between MUP and MKP clinically needless.

## Data Availability Statement

The datasets presented in this study can be found in online repositories. The names of the repository/repositories and accession number(s) can be found below: Del Fiore, Paolo (2020), “Melanoma of Unknown Primary (MUP) Monocentric Retrospectivr Study”, Mendeley Data, V1, doi: 10.17632/xj636ftgff.1.

## Ethics Statement

The studies involving human participants were reviewed and approved by the Ethics Committee of Veneto Institute of Oncology CESC-IOV. The patients/participants provided their written informed consent to participate in this study.

## Author Contributions

Study concepts: PF, MR, SM. MA, VC, JP. Study design: PF, FC, SM, MA, CR. Data acquisition: PF, RS, FC, GA, AP, BF, AS. Quality control of data and algorithms: PF, FC. Data analysis and interpretation: PF, FC, SM, DL, GA. Statistical analysis: FC. Manuscript preparation: PF, FC, SM, AB, RC, GA. Manuscript editing: PF, FC, SM, DL, MR. Manuscript review: SM, AB, AF, FB, MA, RM, CR. All authors contributed to the article and approved the submitted version.

## Conflict of Interest

The authors declare that the research was conducted in the absence of any commercial or financial relationships that could be construed as a potential conflict of interest.
