# Corrigendum: Melanoma of Unknown Primary: Evaluation of the Characteristics, Treatment Strategies, Prognostic Factors in a Monocentric Retrospective Study

**DOI:** 10.3389/fonc.2021.686051

**Published:** 2021-04-14

**Authors:** Paolo Del Fiore, Marco Rastrelli, Luigi Dall’Olmo, Francesco Cavallin, Rocco Cappellesso, Antonella Vecchiato, Alessandra Buja, Romina Spina, Alessandro Parisi, Renzo Mazzarotto, Beatrice Ferrazzi, Andrea Grego, Alessio Rotondi, Clara Benna, Saveria Tropea, Francesco Russano, Angela Filoni, Franco Bassetto, Angelo Paolo Dei Tos, Mauro Alaibac, Carlo Riccardo Rossi, Jacopo Pigozzo, Vanna Chiarion Sileni, Simone Mocellin

**Affiliations:** ^1^ Surgical Oncology Unit, Veneto Institute of Oncology IOV - IRCCS, Padua, Italy; ^2^ Department of Surgery, Oncology and Gastroenterology (DISCOG), University of Padua, Padua, Italy; ^3^ Emergency Department- Azienda Ospedaliera Padova, Padova, Italy; ^4^ Independent Statistician, Solagna, Italy; ^5^ Surgical Pathology and Cytopathology Unit, Department of Medicine (DIMED), University of Padua, Padua, Italy; ^6^ Department of Cardiological, Thoracic, Vascular Sciences and Public Health, University of Padua, Padua, Italy; ^7^ Radiotherapy Unit, Veneto Institute of Oncology, IOV-IRCCS, Padua, Italy; ^8^ Department of Radiotherapy, Ospedale Civile Maggiore, Azienda Ospedaliera Universitaria Integrata, Verona, Italy; ^9^ Postgraduate School of Occupational Medicine, University of Verona, Verona, Italy; ^10^ Department of Medicine (DIMED), University of Padua, Padua, Italy; ^11^ Clinic of Plastic Surgery, Department of Neuroscience, Padua University Hospital, University of Padua, Padua, Italy; ^12^ Unit of Dermatology, University of Padua, Padua, Italy; ^13^ Melanoma Oncology Unit, Veneto Institute of Oncology IOV-IRCCS, Padua, Italy

**Keywords:** melanoma of unknown primary, occult primary melanoma, skin cancer, melanoma, MUP, melanoma treatment, immunotherapy, target therapy

An author name Angelo Paolo Dei Tos was incorrectly spelled in the citation as **“**Tos APD”. The correct spelling is “Dei Tos AP”.

The authors apologize for this error and state that this does not change the scientific conclusions of the article in any way. The original article has been updated.

